# Polarized Distribution of Active Myosin II Regulates Directional Migration of Cultured Olfactory Ensheathing Cells

**DOI:** 10.1038/s41598-017-04914-z

**Published:** 2017-07-05

**Authors:** Cheng-gen Zheng, Fan Zhang, Xiao-mei Bao, Shi-yang Wu, Peng Wang, Jia-nan Zhou, Yuan Gao, Hong-lin Teng, Ying Wang, Zhi-hui Huang

**Affiliations:** 1 Department of Cardiology, Chun’an First People’s Hospital (Zhejiang Province People’s Hospital Chun’an Branch), Hangzhou, 311700 China; 2 Institute of Neuroscience and Institute of Hypoxia Medicine, Wenzhou Medical University, Wenzhou, Zhejiang 325035 China; 3 Department of Transfusion Medicine, Zhejiang Provincial People’s Hospital of Hangzhou Medical College, Hangzhou, 310053 China; 4 Department of Spine Surgery, the First Affiliated Hospital of Wenzhou Medical University, Wenzhou, Zhejiang 325035 China

## Abstract

Migration of olfactory ensheathing cells (OECs) is critical for development of olfactory system and essential for neural regeneration after OEC transplantation into nerve injury site. However, the molecular mechanisms underlying the regulation of directional migration of OECs remain unclear. In this study, we found that in migrating OECs, phosphorylated myosin light chain (p-MLC, active myosin II) displayed a polarized distribution, with the leading front exhibiting higher than soma and trailing process. Over-expression of GFP-MLC significantly reduced OEC migration. Moreover, decreasing this front-to-rear difference of myosin II activity by the frontal application of a ML-7 (myosin II inhibitors) gradient induced the collapse of leading front and reversed soma translocation of OECs, whereas, increasing this front-to-rear difference of myosin II activity by the rear application of a ML-7 or BDM gradient or the frontal application of a Caly (myosin II activator) gradient accelerated the soma translocation of OECs. Finally, myosin II as a downstream signaling of repulsive factor Slit-2 mediated the reversal of soma translocation induced by Slit-2. Taken together, these results suggest that the polarized distribution of active myosin II regulates the directional migration of OECs during spontaneous migration or upon to extracellular stimulation such as Slit-2.

## Introduction

As a unique type of glial cells in the olfactory system, olfactory ensheathing cells (OECs) have been discovered to promote the growth of olfactory sensory axons during development and the regeneration of injured axons after being transplanted into nerve injury sites. These cells share some features and functions with astrocytes and Schwann cells^1, 2^. Unlike other type glia, OECs migrate from the periphery (olfactory epithelium) into the central nervous system (olfactory bulb), and organized OEC migration can enhance axonal extension after injury^3^. During development, derived from the olfactory placode, OECs migrate out of the olfactory epithelium together with growing olfactory sensory axons from the lamina propria, and accumulate as a superficial mass upon reaching the telencephalic vesicle at embryonic day (E) E13-E18 in rats, contributing to the formation of presumptive olfactory nerve layer^3–6^. During this process, OECs pioneer the olfactory nerve pathway and provide a conductive substrate for the growth of primary olfactory axons, and are required for embryonic olfactory axon targeting and the migration of gonadotropin-releasing hormone neurons^7–10^. Stimulation of OEC motility enhances olfactory axon growth^3, 11, 12^. In OEC transplantations, OECs have to migrate from transplanted sites to injury sites to promote neural regeneration. Although some factors have been identified to regulate OEC migration^3, 13–20^, the molecular mechanisms underlying the regulation of directional migration of OECs remain unclear.

Myosin II subfamily belongs to myosin superfamily of actin-based molecular motors with at least 25 different classes^21^. This subfamily includes skeletal, cardiac and smooth muscle myosin, as well as nonmuscle myosin II (NMII), which are the most members. All myosin II molecules are hexamers composed of myosin II heavy chain (MHC) dimers and two pairs of myosin light chains (MLCs). Myosin II can bind reversibly to actin filaments, hydrolyze ATP in a process that is activated by actin and thereby convert chemical energy into mechanical force and movement. The regulation of myosin II activation is through phosphorylation of the 20 kDa MLC^22, 23^. MLC20 is a substrate for a number of kinases, including Ca^2+^-calmodulin-dependent MLC kinase (MLCK)^24^, Rho kinase and AMP-activated protein kinase (AMP kinase)^23, 25^. These kinases phosphorylate MLC20 primarily on Ser19^26^, which increases actin-activated MgATPase activity, filament formation and contractile activity *in vitro* and *in vivo* ^21^. Recent studies have shown that NMII play a critical role in three related cellular activities: generation of cell polarity, cell migration and cell-cell adhesion^21^. However, it remains unknown that whether NMII regulates the directional migration of OECs during spontaneous migration or upon to extracellular factor stimulation.

In the present study, we found that the polarized distribution of active myosin II regulates the directional migration of OECs during spontaneous migration or upon to the repulsive factor Slit-2.

## Results

### The polarized distribution of active myosin II in migrating OECs

**Figure 1:**
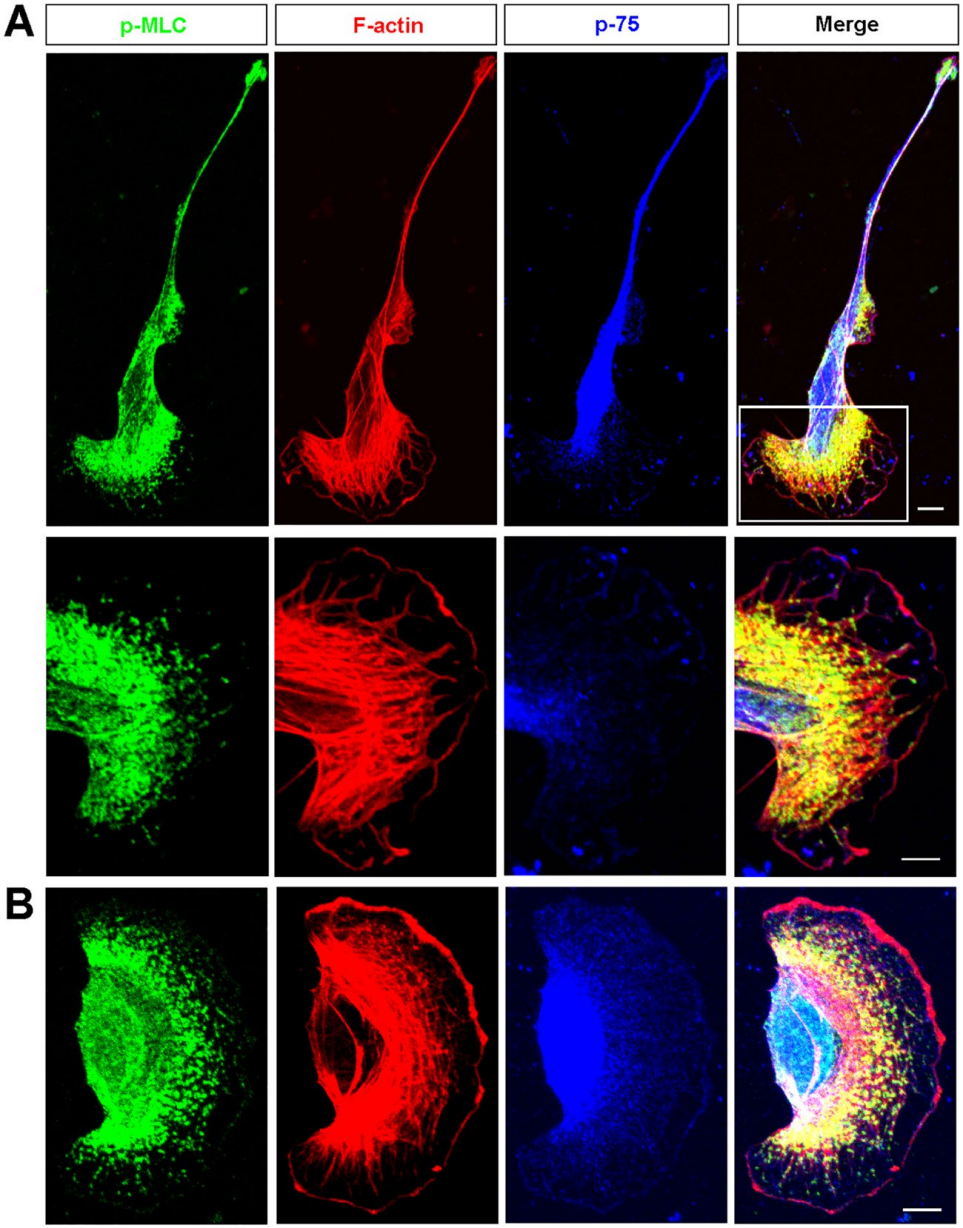
The cellular distribution of p-MLC in OECs. (**A**) Triple immunostaining of p-MLC (green), F-actin (red) and p75 (blue) in Schwann-cell like OECs. Images of selected regions were shown as at higher magnification. (**B**) Triple immunostaining of p-MLC (green), F-actin (red) and p75 (blue) in astrocyte-like OECs. Scale bars, 20 μm.

To explore the potential effects of myosin II in OEC migration, we firstly examined the cellular distribution of active myosin II in cultured OECs. Immunostaining of p-MLC (serine-19, myosin light chain, MLC), which marks the activated form of myosin II, was performed. As shown in Fig. 1A, in Schwann cell-like OECs with higher motility^27^, p-MLC displayed a polarized distribution, with the leading front exhibiting higher than the soma and trailing process. p-MLC mainly distributed at the center of leading front, and partially co-localized with F-actin (Fig. 1A). In another subtype OECs, astrocyte-like OECs, p-MLC displayed similar distribution, mainly at the center of leading front (Fig. 1B).

**Figure 2:**
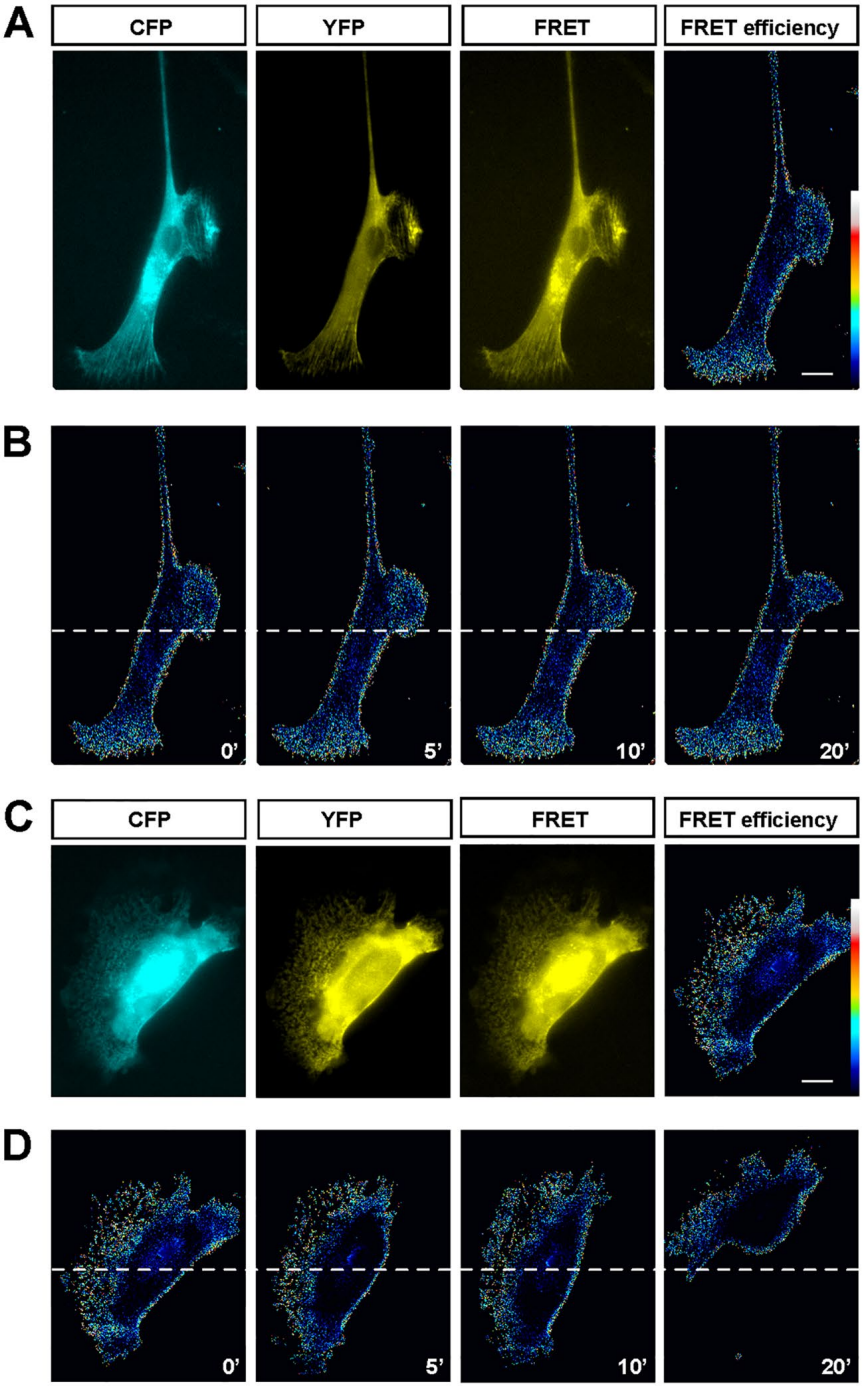
The distribution of active myosin II in migrating OECs. (**A**) Representative FRET images with three-channel microscopy showed the distribution of active myosin II in cultured Schwann-cell like OECs. (**B**) Images showed dynamic FRET signal (in pseudocolors) of active myosin II in the migrating Schwann-cell like OECs. (**C**) Representative FRET images with three-channel microscopy showed the distribution of active myosin II in cultured astrocyte-like OECs. (**D**) Images showed dynamic FRET signal (in pseudocolors) of active myosin II in the migrating astrocyte-like OECs. Scale bars, 20 μm. Time, minutes.

To further examine the distribution of active myosin II in migrating alive OECs, cultured OECs were transfected with a fluorescence resonance energy transfer (FRET)-based myosin II biosensor.As shown in Fig. 2A, in Schwann-cell like OECs, the FRET signal for the active myosin II also displayed a polarized distribution, with the leading front exhibiting higher activity than soma and trailing process. In astrocyte-like OECs, the FRET signal mainly distributed at the center of leading front (Fig. 2C). These results suggest that active myosin II mainly distributes in center of the leading front, but not soma. This notion was further supported by the photometric analysis of the active myosin II in migrating OECs based on time-lapse imaging. As shown in Fig. 2B and D, the active myosin II maintained this polarized distribution, mainly distributed at the center of leading front during OEC migration. Taken together, these results suggest that the polarized distribution of active myosin II may be critical to regulate directional migration of OECs.

### Over-expression of MLC reduced OEC migration

**Figure 3:**
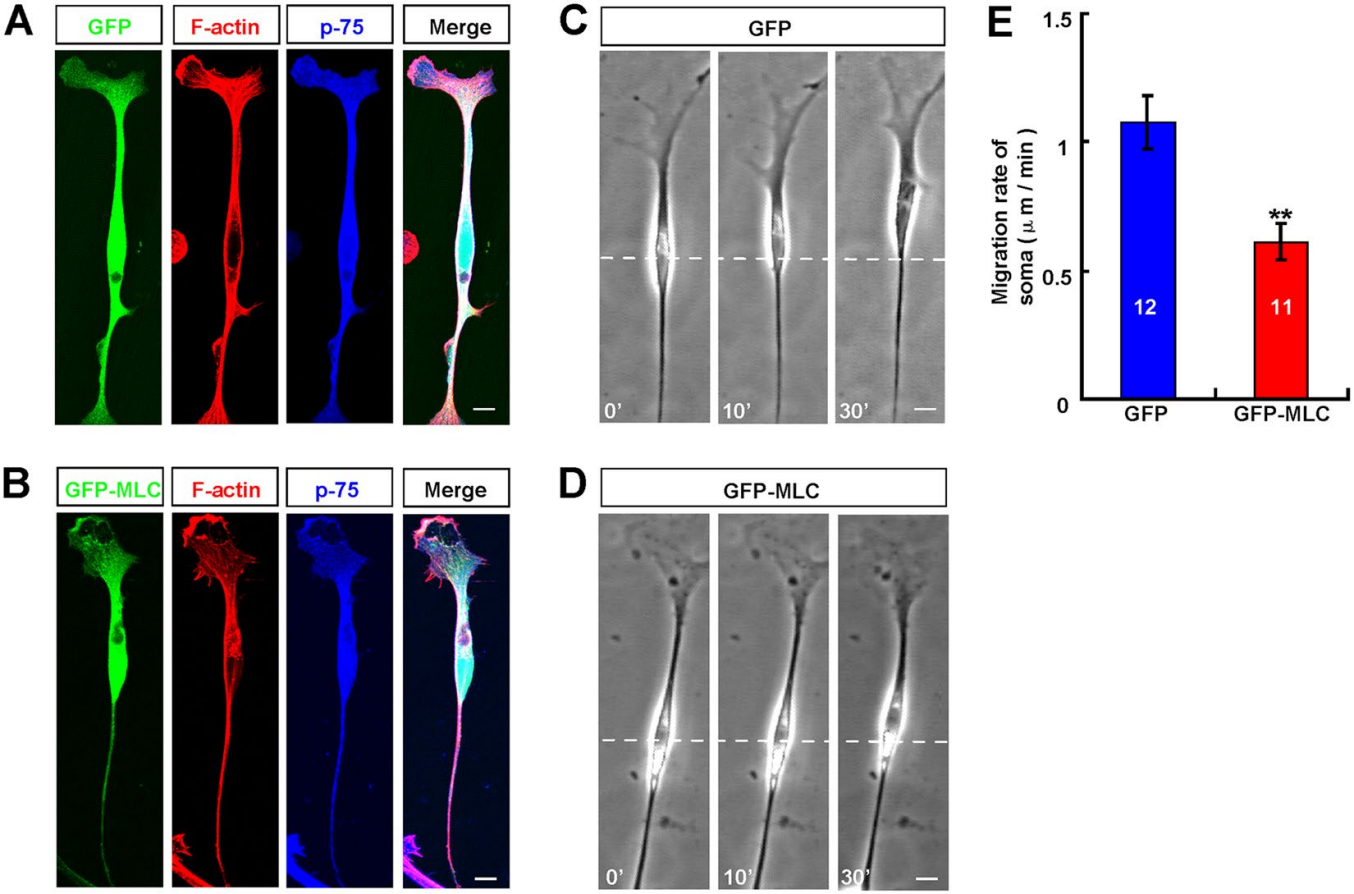
Over-expression of MLC reduced OEC migration. (**A–B**) Double immunostaining of F-actin (red) and p-75 (blue) in OECs transfected with EGFP (**A**) or EGFP-MLC (**B**). (**C–D**) Time-lapse images of migrating OECs transfected with EGFP (**C**) or EGFP-MLC (**D**). (**E**) Quantitative analysis of migration rate of OECs transfected with EGFP or EGFP-MLC. Time, min, scale bar, 20 μm. Data were mean ± SD. ^**^ *P* < *0.01*, compared with control, Student’s t-test.

To examine the role of active myosin II in OEC migration, cultured OECs were ﻿firstly﻿ transfected with GFP-MLC. As shown in Fig. 3A and B, GFP-MLC mainly distributed in the soma and leading front of OECs, partially co-localized with F-actin. Surprisingly, these GFP-MLC OECs migrated more slowly than control GFP OECs (Fig. 3C,D). Indeed, statistical analysis showed that over-expression of GFP-MLC in OECs significantly reduced the motility of OECs, compared with control GFP cells (Fig. 3E). These results suggest over-expressing MLC may disrupt the polarized distribution of active myosin II, and then inhibit OEC migration.

### Decreasing the front-to-rear difference of myosin II activity induced the collapse of lead front and reversal of soma translocation of OECs

**Figure 4:**
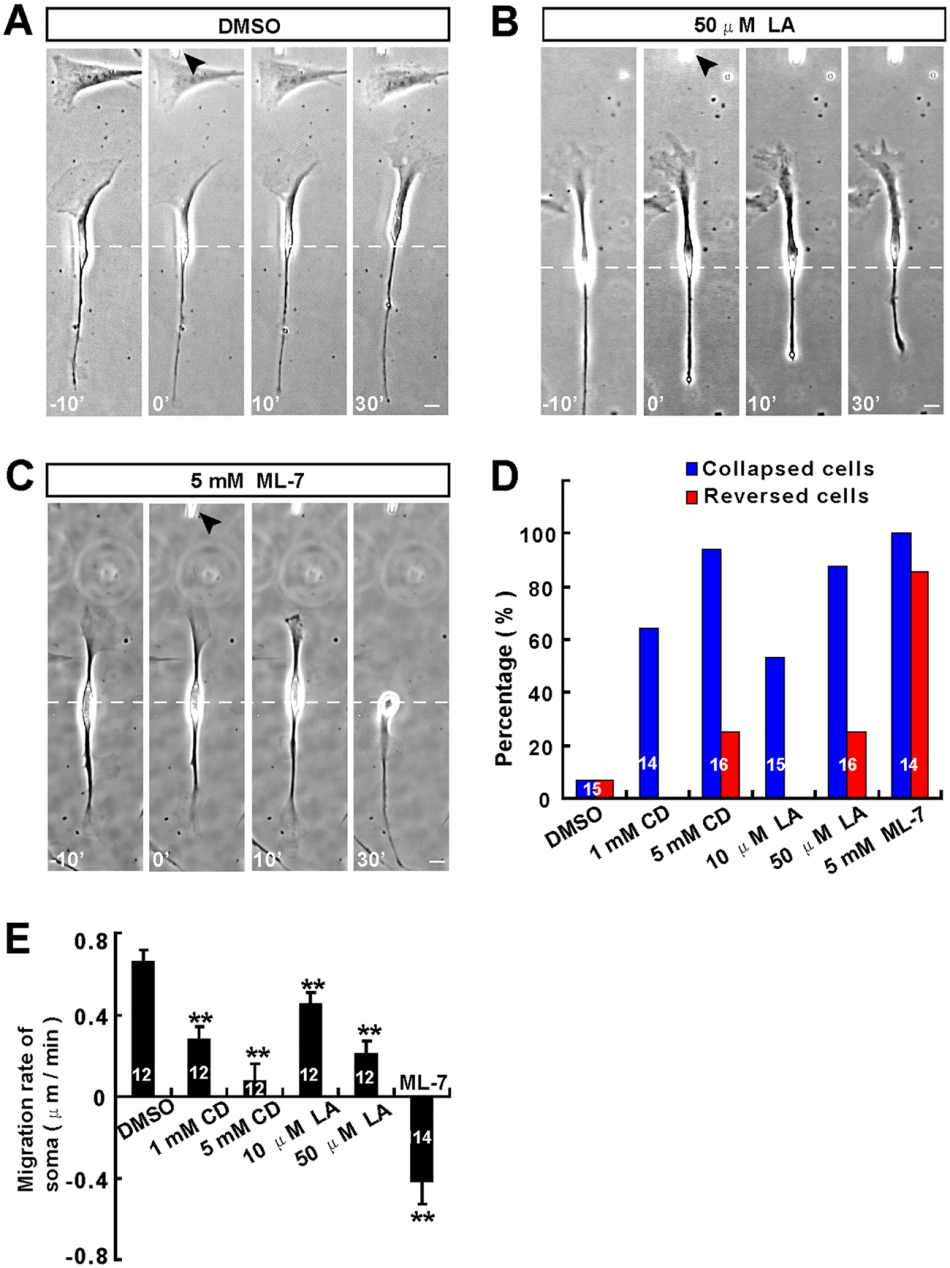
Decreasing the front-to-rear difference of myosin II activity induced the collapse of lead front and reversal of soma translocation of Schwann cell-like OECs. (**A–C**) Images of migrating Schwann cell-like OECs before and after the frontal application of a gradient of DMSO as a control (**A**), latrunculin A (LA, 50 μM in micropipette, **B**) or ML-7 (5 mM in micropipette, **C**). (**D–E**) Summary of the percentages of collapsed or reversed cells in total observed cells (**D**) and migration rates of the soma (**E**) under various conditions. White arrowheads indicated the direction of the micropipette. Time, min; scale bars, 20 μm. Data were mean ± SD. ^**^ *P* < *0.01*, compared with control, Student’s *t*-test.

To further examine whether the polarized distribution of active myosin II determines the direction of OEC migration, OEC single-cell directional migration assay was performed. In this assay, microscopic gradient of migratory cue was produced by repetitive puffing of the solutions containing the candidate factors through a micropipette placed at a distance of 100 μm in front of the soma of migrating OECs to mimic the gradient of migratory factors *in vivo*. Under this standard condition, the concentration of factor at a distance of 100 μm from the micropipette tip was about 10^−3^ fold lower than in the micropipette^28^. We firstly applied a gradient of ML-7 (5 mM in micropipette), a specific inhibitor of the myosin light chain kinase, in front of migrating Schwann cell-like OECs (with higher motility in OEC subtypes) to disrupt the polarized distribution of myosin II activity across the cell. Indeed, ML-7 incubation significantly decreased the p-MLC level in cultured OECs (Supplementary material, Fig. S1). As shown in Fig. 4C, after the application of ML-7 gradient, the leading front was inhibited in their motility and showed collapse and retraction within 10 min. Most of tested cells, the soma later reversed their direction of translocation, with the original trailing tail becoming a new leading front (Fig. 4C,D). As control, application of latrunculin A (LA) or cytochalasin D (CD), inhibitors of F-actin polymerization, induced obvious collapse of leading front within 10 mins (Fig. 4B,D), however, only a few of these cells later reversed their soma translocation (Fig. 4B,D). OECs migration was not affected by the gradient of DMSO, as a control (Fig. 4A,D). Quantitative analysis revealed that the average migration rate of soma significantly was decreased under CD or LA or ML-7 gradient, compared with control (Fig. 4E). These results suggest the polarized distribution of myosin II may determines the direction of Schwann cell-like OECs migration.

**Figure 5:**
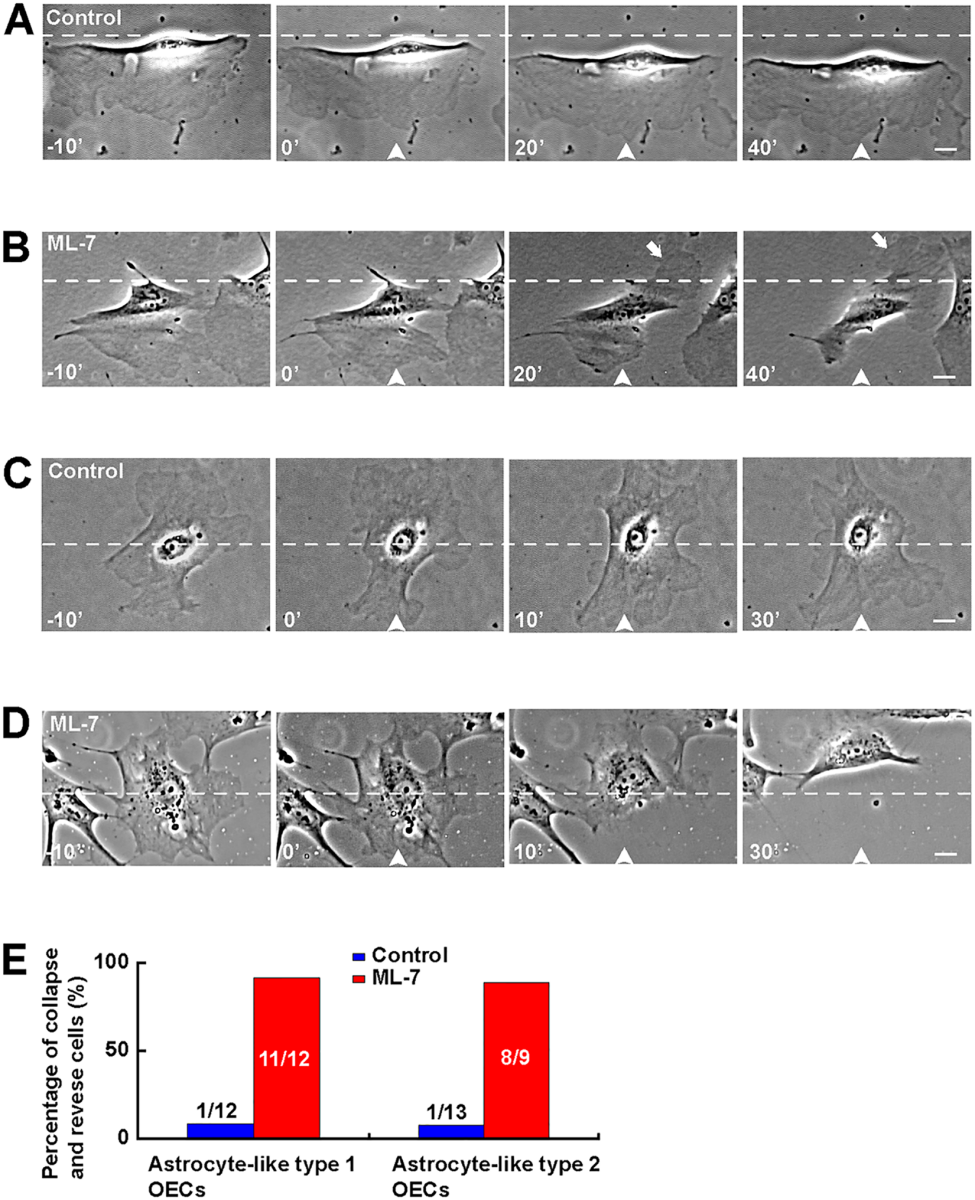
Disruption of active myosin II distribution induced the collapse of leading front and inhibited migration of astrocyte-like OECs. (**A–B**) Images of migrating astrocyte-like type 1 OECs before and after the frontal application of a gradient of DMSO as a control (**A**), or ML-7 (5 mM in micropipette, **B**). (**C–D**) Images of migrating astrocyte-like type 2 OECs before and after the frontal application of a gradient of DMSO as a control (**C**), or ML-7 (5 mM in micropipette, **D**). (**E**) Histogram showing the percentages of collapsed and reversed OECs in total cells after application ML-7. Time, min, scale bar, 20 μm.

We next examined the role of active myosin II in astrocyte-like OECs, which has two subtypes: type 1 OECs and type 2 OECs^27^. Interestingly, after the frontal application of ML-7 gradient, astrocyte-like type 1 OECs was inhibited in their motility and showed obvious collapse within 20 min (Fig. 5B), and the original trailing tail regrew new lamellipodia, became a new leading front (Fig. 5B, white arrow indicated). When ML-7 gradient was applied to astrocyte-like type 2 OECs, these cells also showed obvious collapse, became into astrocyte-like type 1 morphological phenotype, and escaped from the micropipette (Fig. 5D). Astrocyte-like OEC migration was not affected by the gradient of DMSO, as a control (Fig. 5A,C). Quantitative analysis revealed that 91.7% of 12 astrocyte-like type 1 OECs showed collapse and reversed soma translocation under ML-7 gradient, compared with 8.3% of 12 under DMSO control, and 88.9% of 9 astrocyte-like type 2 OECs showed collapse and reversed the soma translocation, compared with 7.7% of 13 astrocyte-like type 2 OECs (Fig. 5E). Taken together, these results suggest active myosin II at leading front of OECs may maintain the morphology and the stability of leading front, and determine the direction of OEC migration.

### Increasing the front-to-rear difference of myosin II activity promoted Schwann cell-like OEC migration

**Figure 6:**
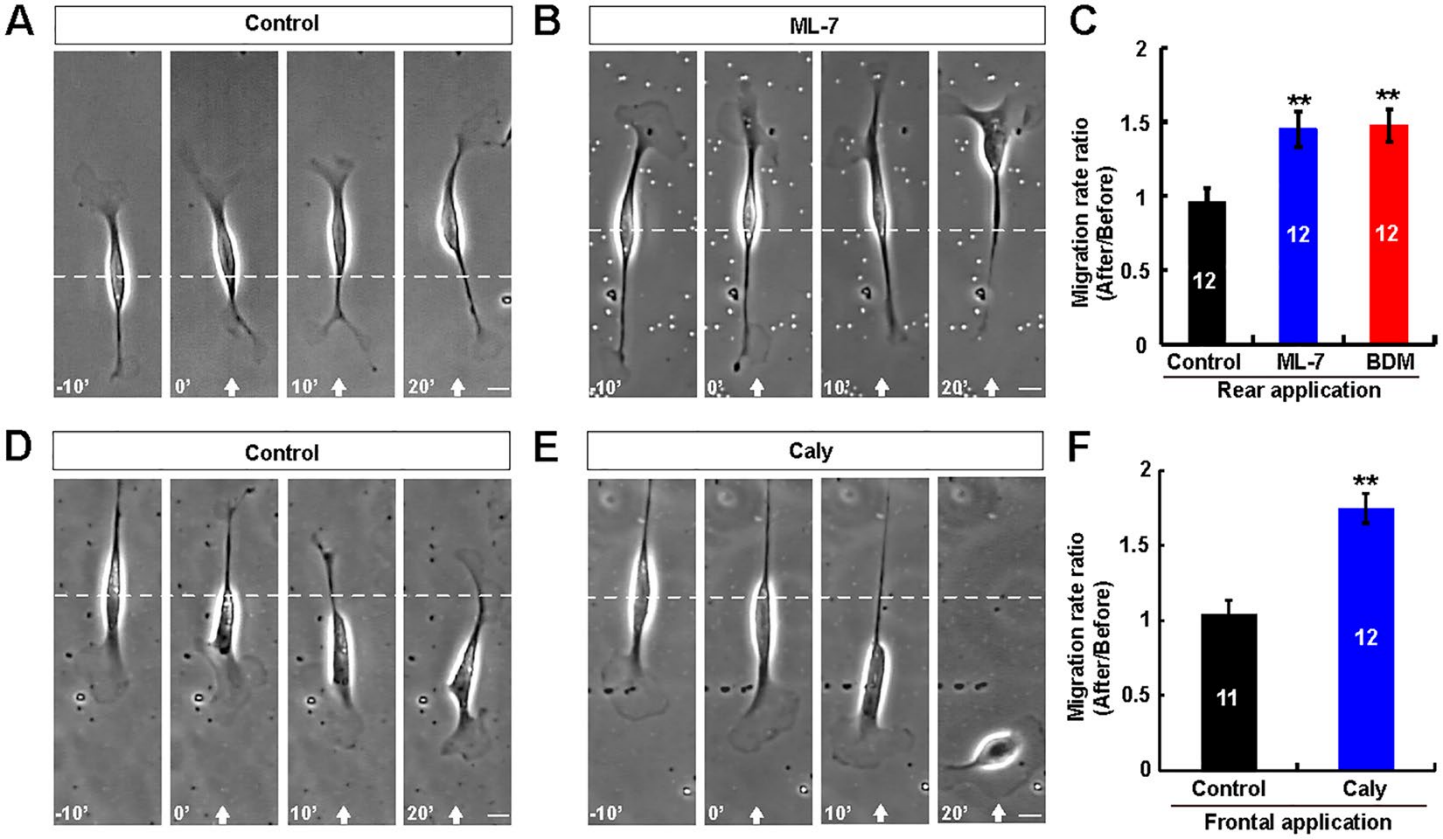
Increasing the front-to-rear difference of myosin II activity accelerated the soma translocation of Schwann cell-like OECs. (**A–B**) Images of migrating Schwann cell-like OECs before and after the rear application of a gradient of DMSO (**A**), or ML-7 (**B**). (**C**) Summary of migration rate ratios (after/before) of soma under the rear application of DMSO, or ML-7 or BDM. (**D–E**) Images of migrating Schwann cell-like OECs before and after the frontal application of a gradient of DMSO (**D**), or Caly (**E**). (**F**) Summary of migration rate ratios (after/before) of soma under the frontal application of DMSO or Caly. White arrowheads indicated the direction of the micropipette. The micropipette was loaded with 5 mM ML-7, or 200 mM BDM, or 25 μM Caly. Time, min; scale bars, 20 μm. Data were mean ± SD. ^**^ *P* < *0.01*, compared with control, Student’s *t*-test.

We next examined whether increasing the front-to-rear difference of myosin II activity could promote the migration of Schwann cell-like OECs. As shown in Fig. 6B, we applied ML-7 or BDM gradient from the rear to decrease of myosin II activity at the trailing process. After the rear application of the ML-7 or BDM gradient, the trailing process showed collapse and retraction, whereas, the soma translocation was accelerated dramatically. Schwann cell-like OEC migration was not affected by DMSO, as a control (Fig. 6A,C). Quantitative analysis revealed that the average migration rate ratio significantly was increased under ML-7 or BDM gradient, compared to control (Fig. 6C). These results suggest that the front-to-rear difference in myosin II activity may mediate the soma translocation of Schwann cell-like OECs. To further support this notion, as shown in Fig. 6E and F, after the frontal application of the phosphatase inhibitor Caly (25 uM in micropipette), which activates myosin II activity by keeping the myosin regulatory light chain phosphorylated, the soma translocation was also accelerated significantly. Taken together, these results further suggest that the polarized distribution of myosin II activity determines the direction of migration Schwann cell-like OECs during spontaneous migration.

### Myosin II as a downstream signaling of repulsive factor Slit-2 mediated the reversal of soma translocation of OECs

**Figure 7:**
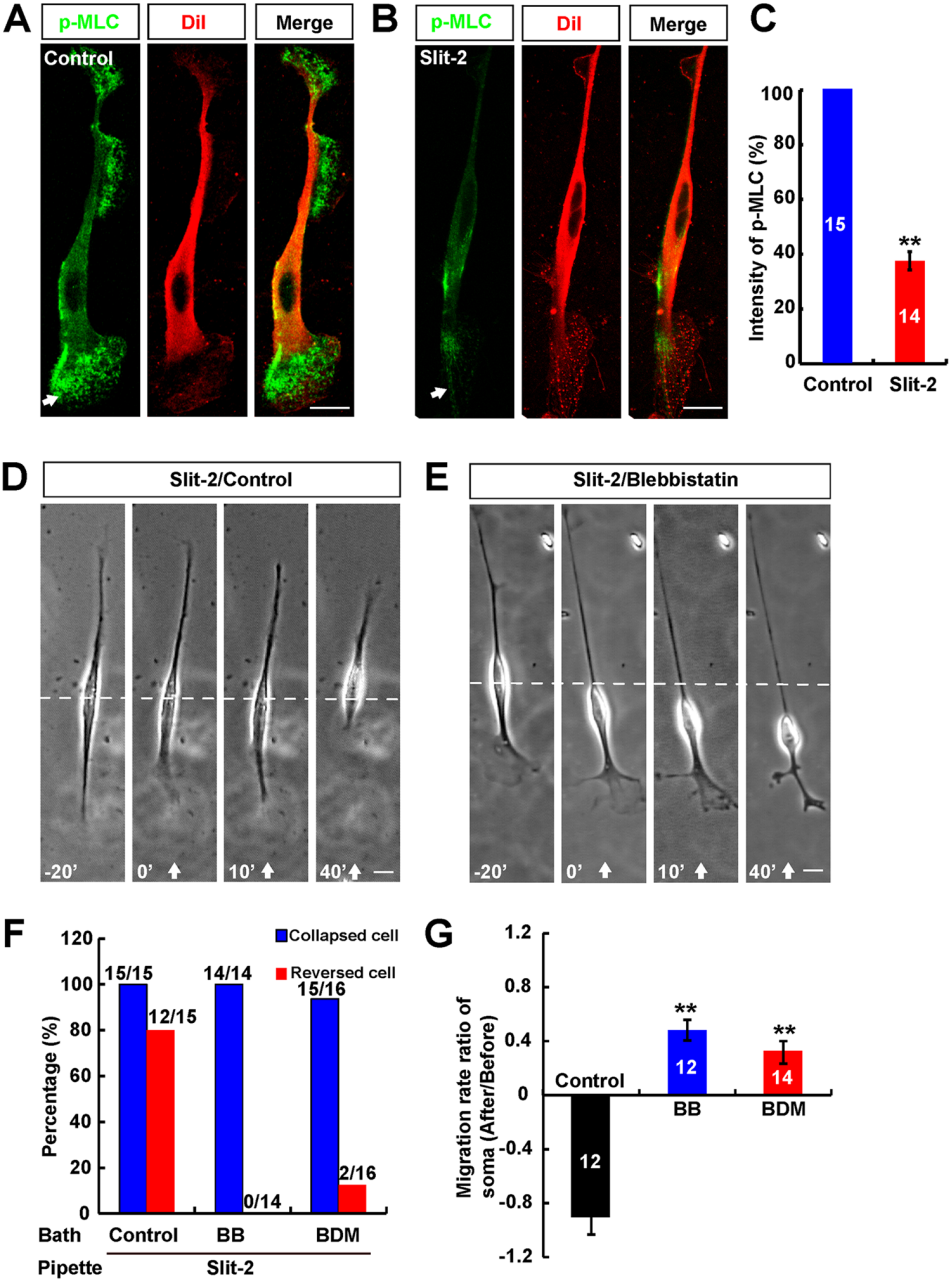
Myosin II as a downstream signaling of repulsive factor Slit-2 mediated the reversal of soma translocation of OECs. (**A–B**) Immunocytochemical analysis of the distribution of p-MLC (green) with (**B**) or without (**A**) Slit-2 treatment. Dil dye (red) is a membrane marker. (**C**) Quantitative analysis of relative p-MLC intensity (normalized to control) at leading front of OECs with or without Slit-2 incubation. (**D–E**) Images of migrating OECs before and after exposure to a Slit-2 gradient with (**D**) or without (**E**) blebbistatin incubation. White arrowheads indicated the direction of micropipette. (**F–G**) Summary of the percentages of collapsed or reversed cells in total observed OECs (**F**) and average rate migration ratios of soma (**G**) in response to Slit-2 gradient under various conditions. Data were mean ± SD. ^**^ *P* < *0.01*, compared with control﻿, Student’s *t*-test. Scale bars, 20 μm.

Our previous studies have shown that repulsive factor Slit-2 induces collapse of leading process and reversal of soma translocation of OECs^13^. Since myosin II activity plays a critical role in the soma translocation of OECs, we next examined whether myosin II activity as a downstream of Slit-2 mediated the reversal of soma translocation in OECs. We first observed whether Slit-2 treatment could inactivate myosin II in OECs by immunostaining. Meanwhile, we labeled the plasma membrane by using membrane marker Dil dye (1, 1′–dioctadecyl–3, 3, 3′ 3′–tetramethylindocarbocyanine) to observe the relative distribution of p-MLC in OECs. Again, as shown in Fig. 7A, in control OECs, p-MLC displayed a polarized distribution, with the leading front higher than the soma and trailing tail. After Slit-2 incubation, p-MLC in the whole OECs dramatically decreased, especially, at leading front (Fig. 7B). Quantitative analysis revealed that p-MLC in OECs was significantly decreased by Slit-2, compared with control (Fig. 7C). These results suggest that Slit-2 incubation inactivates myosin II.

We next examined the roles of myosin II in Slit-2-induced OEC migration. OECs were pretreated with specific inhibitors of myosin II, blebbistatin (BB), or 2, 3-butanedione monoxime (BDM) in bath for at least 30 mins. As shown in Fig. 7E,F, the frontal application of Slit-2 gradient to these BB or BDM-OECs still induced the collapse of leading front, but failed to reverse soma translocation. In contrast, pretreatment with DMSO as a control in bath did not affect Slit-2-induced repulsive migration of OECs (Fig. 7D,F). Quantitative analysis revealed that the average migration rate ratio of soma significantly was decreased after BB or BDM pretreatment in bath, compared with control (Fig. 7G). Taken together, these results suggest that myosin II signaling is required for the reversal of soma translocation induced by Slit-2 in OECs.

## Discussion

In this study, we present evidence for myosin II’s function in OEC migration. During spontaneous migration, front-to-rear polarized distribution of active myosin II determines the directional migration of OECs, whereas, upon to repulsive factor Slit-2, switch from front-to-rear to rear-to front polarized distribution of active myosin II may be required for Slit-2-induced reversal of soma translocation.

OEC transplantation has emerged as a very promising experimental therapy to treat the axonal injuries of central nervous system^29–36^. Organized migration of OECs enhances the axon regeneration^3, 11, 12^. However, it remains unclear that molecular mechanism underlying OEC migration. Cell migration is a highly regulated and coordinated process, comprised of several steps that include polarization, protrusion, and adhesion formation and turnover at the cell front, along with adhesion disassembly and tail retraction at the cell rear. Much is known about some of these processes, however, less is known about their polarization. In epithelial cells and astrocytes, polarity is established through a signaling pathway involving Cdc42/aPKC/GSK/PAR6 pathway that orients the MTOC, Golgi apparatus, and nucleus^37–39^. In fibroblasts, activated myosin II creates a region of actomyosin filament bundles that terminate in adhesions^40, 41^. Our previous studies have found that during spontaneous migration, OECs can polarize spontaneously and migrate in a directionally persistent manner^27^, and overexpression of DN-Cdc42 did not affect spontaneous migration of OECs^13^, which suggest that Cdc42 pathway is not required for directional migration of OECs. Although OECs share with some features with astrocytes, their mechanism of spontaneous migration may be different.

Recent studies have reported that in cultured cerebellar granule cells or Schwann cells, myosin II activity is enriched in the proximal region of leading front, and is responsible for the coordinated motility of the centrosome and soma^42, 43^, generating the traction force along the leading process that drives the forward translocation of the soma^44^. In the present study, there are several lines of evidences suggest a critical role for myosin II in regulating the directional migration of OECs. First, consistent with these findings, we also found the polarized distribution of active myosin II in migrating OECs. Second, overexpression of GFP-MLC could dramatically reduce the migration of OECs, which may be due to disruption of endogenous p-MLC distribution. Third, decreasing or increasing the front-to-rear difference of myosin II activity reversed or accelerated soma translocation. Interestingly, inhibition of myosin II activity at leading front by ML-7 or BDM gradient induced the collapse and retraction of leading front, and reversed the soma translocation. These results are consistent with previous study that ML-7 induced the retraction of mature oligodendrocyte processes^45^, however, different to the studies in neuron, that the frontal application of myosin II inhibitor in migrating neuron only suppresses the soma translocation, not affect the leading front^44^. These results suggest that different functions of myosin II exist in neuron and glia cells. Interestingly, the collapse of leading front by F-actin polymerization inhibitors LA or CD only inhibited the soma translocation, whereas the collapse of leading front by ML-7 or BDM could reverse soma translocation. These results suggest that myosin II not only is involved in the lamellipodia extension of leading front, but also be essential for the re-orientation and stabilization of the direction of soma translocation of OECs.

Whether myosin II is also involved in directional migration upon to some guidance factors? Our previous studies have shown that repulsive factor Slit-2 induces the collapse of leading front and reverses the soma translocation in OECs^13^. In the present study, there are several lines of evidences suggesting a critical role for myosin II in Slit-2-induced OECs. First, incubation of Slit-2 inactivated myosin II in OECs, especially at the leading front. Second, Slit-2-induced reversal of soma translocation was blocked by myosin II inhibitors in bath. Myosin II is known to be activated by Rho kinase^21^. Our previous studies have shown that calcium-RhoA-Rock signaling is involved in the reversal of soma translocation induced by Slit-2 in OECs^13^, and myosin II is required for Slit-2-induced migration of Schwann cells^46^. Recent studies also have shown that Slit-2/Robo2 system inhibits nonmuscle mysosin IIA (NMIIA) activity and destabilizes kidney podocyte adhesion through forming Robo2/SRGAP1/NMIIA complex^47^. Consistent with these studies, thus we propose that myosin II may be a downstream pathway of Slit-2/Robos/RhoA-Rock, to mediate the reversal of soma translocation of OECs induced by Slit-2. How myosin II regulates the Slit-2-induced soma translocation of OECs needs further studies in future.

In summary, we presented evidence to support that the polarized distribution of active myosin II regulates the directional migration of OECs during spontaneous migration or upon to the repulsive factor Slit-2. This knowledge will be helpful for us to better understand the mechanism of OEC migration during development of olfactory system and neural regeneration after nerve injury.

## Methods

### Primary culture and purification of OECs

Primary OEC cultures were prepared from olfactory bulb of adult Sprague-Dawley rats and purified by differential cell adhesiveness as described previously^48, 49^. The authors stated that all experimental methods involving rats were carried out in accordance with relevant guidelines and regulations of the Animal Bioethics Committee of Wenzhou Medical University, and all experimental protocols involving rats were approved by Animal Bioethics Committee of Wenzhou Medical University. Briefly, the meninges were carefully removed from the olfactory bulb under the dissecting microscope and the olfactory nerve layer was peeled away from the glomerular and deeper layers of the olfactory bulb, then dissociated with 0.125% trypsin (Sigma, St Louis, MO) and incubated at 37 °C for 15 min. Trypsinization was stopped by DMEM/F12 (1:1, vol/vol, Gibco, Grand Island, NY) containing 10% heat-inactivated fetal bovine serum (FBS; Hyclone, Logan, UT). The tissue was centrifuged for 10 min at 500 g, and the pellet was triturated using a flame-polished Pasteur pipette and plated on uncoated 25 cm^2^ culture flask (Corning, LY) two times, each for 36 h at 37 °C in 5% CO_2._ The non-adhesive cell suspension was collected and then seeded onto 12-well plates (Corning) pre-coated with poly-L-lysine (PLL; 0.1 mg/ml, Sigma), and incubated with DMEM/F-12 containing 10% FBS, 2 μM forskolin (Sigma) and 10 ng/ml bFGF (R&D systems, MN) as mitogen. The media were changed every 3 days. The overall purity of OECs was around 98%. The definition of OECs is described previously^27, 50^.

### FRET-based imaging of active myosin II with three-channel microscopy

The fluorescence resonance energy transfer (FRET) probe myosin II for monitoring the sub-cellular myosin II activity was kindly provided by Dr. Xiao-bing Yuan (Institute of Neuroscience, CAS, Shanghai). Cells transfected with the FRET probe were imaged on a Nikon Ti microscope with a 40 oil lens (N.A. 1.30) using the Perfect Focus System and were illuminated by a polychrome IV monochromator (TILL Photonics). Filter sets for FRET imaging are CFP (excitation 436 nm, emission, 480/40 HQ, DM 455), FRET (excitation 436 nm, emission, 535/30 HQ, DM 515) and YFP (excitation 510 nm, emission, 535/30 HQ, DM 515). Images of the three channels were recorded simultaneously by using the Cascade 512B CCD (Roper Scientific). Background images were subtracted from the raw images before carrying out FRET calculation. Corrected FRET (FRET^C^) was calculated on a pixel-by-pixel basis for the image using the following equation: FRET^C^ = FRET − a × YFP − b × CFP, where FRET, CFP and YFP corresponded to background-subtracted images, acquired through the FRET, CFP and YFP channels, respectively. “a” and “b” were the fraction of bleed-through of YFP and CFP fluorescence through the FRET channel, respectively, and the two values were determined by using cells transfected with YFP or CFP alone. We used the following equation: E = FRET^C^ /(CFP + FRET^C^) × 100% to quantify the FRET signal by using MetaFluo and Image J software (PixFRET Plug-in)^13, 51–53^.

### Single-cell migration assay based on time-lapse imaging

Single-cell migration assay was described previously^13, 27, 54, 55^. In brief, the purified OECs were re-plated onto square coverslips (8 mm) coated with laminin (10 μg/ml) at a low density of about 1000 cells per coverslip. At 24 h after plating, coverslips with cells were put into a chamber containing 1 ml serum-free L15 medium. The chamber was then covered with a thin layer of methyl-siloxane fluid to prevent evaporation. The experiments were carried out at the heated stage (37 °C) of a phase contrast microscope (CK40, Olympus Optial, Tokyo, Japan). Cells with typical morphology of OECs that were not attached to any other cells were selected. Micropipettes used in pulsatile ejection were pulled with a two-stage puller designed for making patch-clamp electrodes. A micropipette with a tip opening of about 1 μm was placed 15 μm perpendicular and 100 μm away from the center of cell under test. A standard pressure pulse of 3 psi (1 psi = 6.89 kPa) in amplitude and 20 ms in duration was generated by a pulse generator and applied to the pipette at a frequency of 2 Hz. Under this standard condition, the concentration of factor at 100 μm from pipette tip is about 10^−3^ fold lower than that in the pipette^28^. Images of the migrating OECs were recorded, in a time-lapse mode (one picture every 5 min interval), with a CCD camera (JVC TK-1381, Japan) attached to the microscope, and were then stored in a computer for further analysis using Scion imaging software (Frederick, MD). Briefly, we measured the distance of cell migration during a control period and after treatment, and calculated the respective migration rates (distance/time).

### Immunocytochemistry

In brief, the purified OECs were re-plated onto the square coverslips (8 mm) coated with laminin (10 μg/ml, Sigma) at a density of 1000 cells per coverslip or sciatic nerves explants, fixed with fresh 4% paraformaldehyde in 0.1 M PBS (pH 7.4) for 20 min after culturing for 24 h. After washing with PBS, cells were permeabilized with 0.2% Triton X-100 in 0.1 M PBS for 5 min, followed by incubation in blocking buffer (5% normal goat serum and 0.2% Triton X-100 in 0.1 M PBS, pH 7.4) for 1 h, and incubated overnight at 4 °C with polyclonal antibodies against p-MLC (1:50, Cell Signaling Technology); with a monoclonal anti-p-75 antibody (1:500, Chemicon) or diluted in the blocking buffer. Cells were washed three times with PBS and incubated for 1 h at room temperature with an appropriate fluorescence-conjugated secondary antibody (1:1000, Molecular probe, Eugene, OR), and then visualized using confocal or fluorescence microscopy (FV1000, Olympus). No positive signal was observed in control incubations using no primary antibody. For visualization of F-actin, cells were incubated with rhodamine-conjugated phalloidin (1:60, Molecular probe) at room temperature for 1 hour.

For Dil label, OECs were fixed with fresh 4% paraformaldehyde in 0.1 M PBS (pH 7.4) for 20 min. After washing with PBS, cells were incubated for 30 min at room temperature with Dil dye (10 μM, Molecular probe). Washing with PBS three times, cells were then visualized by confocal or fluorescence microscopy (FV1, 000, Olympus).

### Sources and preparation of reagents

2, 3-butanedione monoxime (BDM), Cytochalasin D (CD), latrunculin A (LA) and ML-7 were from Sigma-Aldrich. Caly was from Millipore. Pharmacological agents were dissolved in DMSO or PBS in stock solution. 1 or 5 mM CD or 5 mM ML-7 or 200 mM BDM or 10 or 50 μM LA 25 μM Caly was loaded into the micropipette for migration assay.

### Statistical analysis

All data presented represent results from at least three independent experiments. Statistical analysis was performed using Student’s *t*-test, or using an ANOVA with pair-wise comparisons. Statistical significance was defined as *P < *0.05.

## Electronic supplementary material

Supplementary information

## References

[1] Vincent AJ, West AK, Chuah MI (2005). Morphological and functional plasticity of olfactory ensheathing cells. J Neurocytol.

[2] Higginson JR, Barnett SC (2011). The culture of olfactory ensheathing cells (OECs)–a distinct glial cell type. Exp Neurol.

[3] Ekberg JA, Amaya D, Mackay-Sim A, St John JA (2012). The migration of olfactory ensheathing cells during development and regeneration. Neuro-Signals.

[4] Valverde F, Santacana M, Heredia M (1992). Formation of an olfactory glomerulus: morphological aspects of development and organization. Neuroscience.

[5] Chuah MI, West AK (2002). Cellular and molecular biology of ensheathing cells. Microsc Res Tech.

[6] Katoh H (2011). The dual origin of the peripheral olfactory system: placode and neural crest. Molecular brain.

[7] Tennent R, Chuah MI (1996). Ultrastructural study of ensheathing cells in early development of olfactory axons. Brain Res Dev Brain Res.

[8] Tisay KT, Key B (1999). The extracellular matrix modulates olfactory neurite outgrowth on ensheathing cells. J Neurosci.

[9] Barraud, P., St John, J.A., Stolt, C.C., Wegner, M. & Baker, C.V. Olfactory ensheathing glia are required for embryonic olfactory axon targeting and the migration of gonadotropin-releasing hormone neurons. *Biology open* **2**, 750-759 (2013). 10.1242/bio.20135249 PMC3711043 23862023

[10] Geller S, Kolasa E, Tillet Y, Duittoz A, Vaudin P (2013). Olfactory ensheathing cells form the microenvironment of migrating GnRH-1 neurons during mouse development. Glia.

[11] Windus LC (2011). Stimulation of olfactory ensheathing cell motility enhances olfactory axon growth. Cell Mol Life Sci.

[12] Chehrehasa F (2010). Olfactory glia enhance neonatal axon regeneration. Mol Cell Neurosci.

[13] Huang ZH (2011). Slit-2 repels the migration of olfactory ensheathing cells by triggering Ca2 + -dependent cofilin activation and RhoA inhibition. J Cell Sci.

[14] Su Z (2007). Nogo enhances the adhesion of olfactory ensheathing cells and inhibits their migration. J Cell Sci.

[15] Cao L (2006). Glial cell line-derived neurotrophic factor promotes olfactory ensheathing cells migration. Glia.

[16] Reginensi D (2015). Increased migration of olfactory ensheathing cells secreting the Nogo receptor ectodomain over inhibitory substrates and lesioned spinal cord. Cellular and molecular life sciences: CMLS.

[17] Yan H, Lu D, Rivkees SA (2003). Lysophosphatidic acid regulates the proliferation and migration of olfactory ensheathing cells *in vitro*. Glia.

[18] Wang Y (2016). Brain-derived Neurotrophic Factor Promotes the Migration of Olfactory Ensheathing Cells Through TRPC Channels. Glia.

[19] Ingram NT, Khankan RR, Phelps PE (2016). Olfactory Ensheathing Cells Express alpha7 Integrin to Mediate Their Migration on Laminin. PLoS One.

[20] Nocentini S (2012). Myelin-associated proteins block the migration of olfactory ensheathing cells: an *in vitro* study using single-cell tracking and traction force microscopy. Cellular and molecular life sciences: CMLS.

[21] Conti MA, Adelstein RS (2008). Nonmuscle myosin II moves in new directions. J Cell Sci.

[22] Adelstein RS, Conti MA (1975). Phosphorylation of platelet myosin increases actin-activated myosin ATPase activity. Nature.

[23] Amano M (1996). Phosphorylation and activation of myosin by Rho-associated kinase (Rho-kinase). J Biol Chem.

[24] Nishikawa M, Tanaka T, Hidaka H (1980). Ca2 + -calmodulin-dependent phosphorylation and platelet secretion. Nature.

[25] Nishikawa M, de Lanerolle P, Lincoln TM, Adelstein RS (1984). Phosphorylation of mammalian myosin light chain kinases by the catalytic subunit of cyclic AMP-dependent protein kinase and by cyclic GMP-dependent protein kinase. J Biol Chem.

[26] Ikebe M (1989). Phosphorylation of a second site for myosin light chain kinase on platelet myosin. Biochemistry.

[27] Huang ZH (2008). Migratory properties of cultured olfactory ensheathing cells by single-cell migration assay. Cell Res.

[28] Lohof AM, Quillan M, Dan Y, Poo MM (1992). Asymmetric modulation of cytosolic cAMP activity induces growth cone turning. J Neurosci.

[29] Raisman G, Li Y (2007). Repair of neural pathways by olfactory ensheathing cells. Nat Rev Neurosci.

[30] Chiu SC, Hung HS, Lin SZ, Chiang E, Liu DD (2009). Therapeutic potential of olfactory ensheathing cells in neurodegenerative diseases. J Mol Med.

[31] Cao L (2004). Olfactory ensheathing cells genetically modified to secrete GDNF to promote spinal cord repair. Brain.

[32] Li Y, Field PM, Raisman G (1998). Regeneration of adult rat corticospinal axons induced by transplanted olfactory ensheathing cells. J Neurosci.

[33] Ramon-Cueto A, Plant GW, Avila J, Bunge MB (1998). Long-distance axonal regeneration in the transected adult rat spinal cord is promoted by olfactory ensheathing glia transplants. J Neurosci.

[34] Su Z, He C (2010). Olfactory ensheathing cells: biology in neural development and regeneration. Prog Neurobiol.

[35] Mackay-Sim A, St John JA (2011). Olfactory ensheathing cells from the nose: clinical application in human spinal cord injuries. Exp Neurol.

[36] Khankan RR (2016). Olfactory Ensheathing Cell Transplantation after a Complete Spinal Cord Transection Mediates Neuroprotective and Immunomodulatory Mechanisms to Facilitate Regeneration. The Journal of neuroscience: the official journal of the Society for Neuroscience.

[37] Etienne-Manneville S, Manneville JB, Nicholls S, Ferenczi MA, Hall A (2005). Cdc42 and Par6-PKCzeta regulate the spatially localized association of Dlg1 and APC to control cell polarization. J Cell Biol.

[38] Etienne-Manneville S, Hall A (2002). Rho GTPases in cell biology. Nature.

[39] McCaffrey, L. M. & Macara, I. G. Signaling pathways in cell polarity. *Cold Spring Harbor perspectives in biology* **4** (2012). 10.1101/cshperspect.a009654 PMC3367552 22553378

[40] Vicente-Manzanares M, Koach MA, Whitmore L, Lamers ML, Horwitz AF (2008). Segregation and activation of myosin IIB creates a rear in migrating cells. J Cell Biol.

[41] Vicente-Manzanares M, Newell-Litwa K, Bachir AI, Whitmore LA, Horwitz AR (2011). Myosin IIA/IIB restrict adhesive and protrusive signaling to generate front-back polarity in migrating cells. J Cell Biol.

[42] Solecki DJ (2009). Myosin II motors and F-actin dynamics drive the coordinated movement of the centrosome and soma during CNS glial-guided neuronal migration. Neuron.

[43] Wang Y, Teng HL, Huang ZH (2012). Intrinsic migratory properties of cultured Schwann cells based on single-cell migration assay. PLoS One.

[44] He M, Zhang ZH, Guan CB, Xia D, Yuan XB (2010). Leading tip drives soma translocation via forward F-actin flow during neuronal migration. J Neurosci.

[45] Thomas MG, Santa Coloma TA, Correale J, Boccacci GL (2002). Myosin light chain kinase inhibitors induce retraction of mature oligodendrocyte processes. Neurochem Res.

[46] Wang Y, Teng HL, Huang ZH (2013). Repulsive migration of Schwann cells induced by Slit-2 through Ca2 + -dependent RhoA-myosin signaling. Glia.

[47] Fan X (2016). SLIT2/ROBO2 signaling pathway inhibits nonmuscle myosin IIA activity and destabilizes kidney podocyte adhesion. JCI insight.

[48] Barnett SC, Roskams AJ (2002). Olfactory ensheathing cells. Isolation and culture from the rat olfactory bulb. Methods Mol Biol.

[49] Huang ZH, Wang Y, Yuan XB, He C (2011). RhoA-ROCK-Myosin pathway regulates morphological plasticity of cultured olfactory ensheathing cells. Experimental cell research.

[50] Vincent AJ, West AK, Chuah MI (2003). Morphological plasticity of olfactory ensheathing cells is regulated by cAMP and endothelin-1. Glia.

[51] Feige JN, Sage D, Wahli W, Desvergne B, Gelman L (2005). PixFRET, an ImageJ plug-in for FRET calculation that can accommodate variations in spectral bleed-throughs. Microsc Res Tech.

[52] Fu G (2006). Detection of constitutive heterodimerization of the integrin Mac-1 subunits by fluorescence resonance energy transfer in living cells. Biochem Biophys Res Commun.

[53] Zhang F (2009). Detection of homo- or hetero-association of Doks by fluorescence resonance energy transfer in living cells. Mol Imaging Biol.

[54] Guan CB, Xu HT, Jin M, Yuan XB, Poo MM (2007). Long-range Ca2 + signaling from growth cone to soma mediates reversal of neuronal migration induced by slit-2. Cell.

[55] Huang ZH, Wang Y, Yuan XB, He C (2011). RhoA-ROCK-Myosin pathway regulates morphological plasticity of cultured olfactory ensheathing cells. Exp Cell Res.

