# Supplementary material for: Polarized Distribution of Active Myosin II Regulates Directional Migration of Cultured Olfactory Ensheathing Cells
Source: Sci Rep. 2017 Jul 5;7:4701. doi: 10.1038/s41598-017-04914-z (PMC5498622; doi:10.1038/s41598-017-04914-z)
Supplement: Supplementary file 1 — Supplementary information [file 41598_2017_4914_MOESM1_ESM.pdf]

Polarized Distribution of Active Myosin II Regulates Directional Migration of Cultured Olfactory Ensheathing Cells

Cheng-gen Zheng[1], Fan Zhang[2], Xiao-mei Bao[2], Shi-yang Wu[4], Peng Wang[4], Jia-nan Zhou[2], Yuan Gao[2], Hong-lin Teng[4‡], Ying Wang[1, 2, 3‡], Zhi-hui Huang [2‡]

1. Department of Cardiology, Chun'an First People's Hospital (Zhejiang Province People's Hospital Chun'an Branch), Hangzhou, 311700, China.
2. Institute of Neuroscience and Institute of Hypoxia Medicine, Wenzhou Medical University, Wenzhou, Zhejiang, 325035, China.
3. Department of Transfusion Medicine, Zhejiang Provincial People's Hospital of Hangzhou Medical College, Hangzhou, 310053, China.
4. Department of Spine Surgery, the First Affiliated Hospital of Wenzhou Medical University, Wenzhou, Zhejiang, 325035, China.

‡ Correspondence should be addressed to:

Ying Wang, Email: nancywangying@163.com.

Hong-lin Teng, Email: honlinten@163.com.

Zhi-hui Huang Ph.D, Tel: +86-0577-8699117, Email: hzhzju021@163.com.

Supplementary material

Figure S1. ML-7 treatment significantly decreased p-MLC level in cultured OECs. (**A**) Immunocytochemical analysis of p-MLC (green), F-actin (red) and p-75 (blue) in cultured OECs with or without 5 μM ML-7 incubation for 30 mins. (**B**) Quantitative analysis of the relative p-MLC intensity (normalized to control) in OECs with or without ML-7 incubation as shown in (A). (**C**) Western blot detected the p-MLC level in cultured OECs after 5 μM ML-7 incubation for 30 mins. (**D**) Quantitative analysis of the relative p-MLC level (normalized to control) in OECs after ML-7 incubation as shown in (C). Data were mean ± SD. $^{**}P < 0.01$, Student's *t*-test. Scale bars, 20 μm.

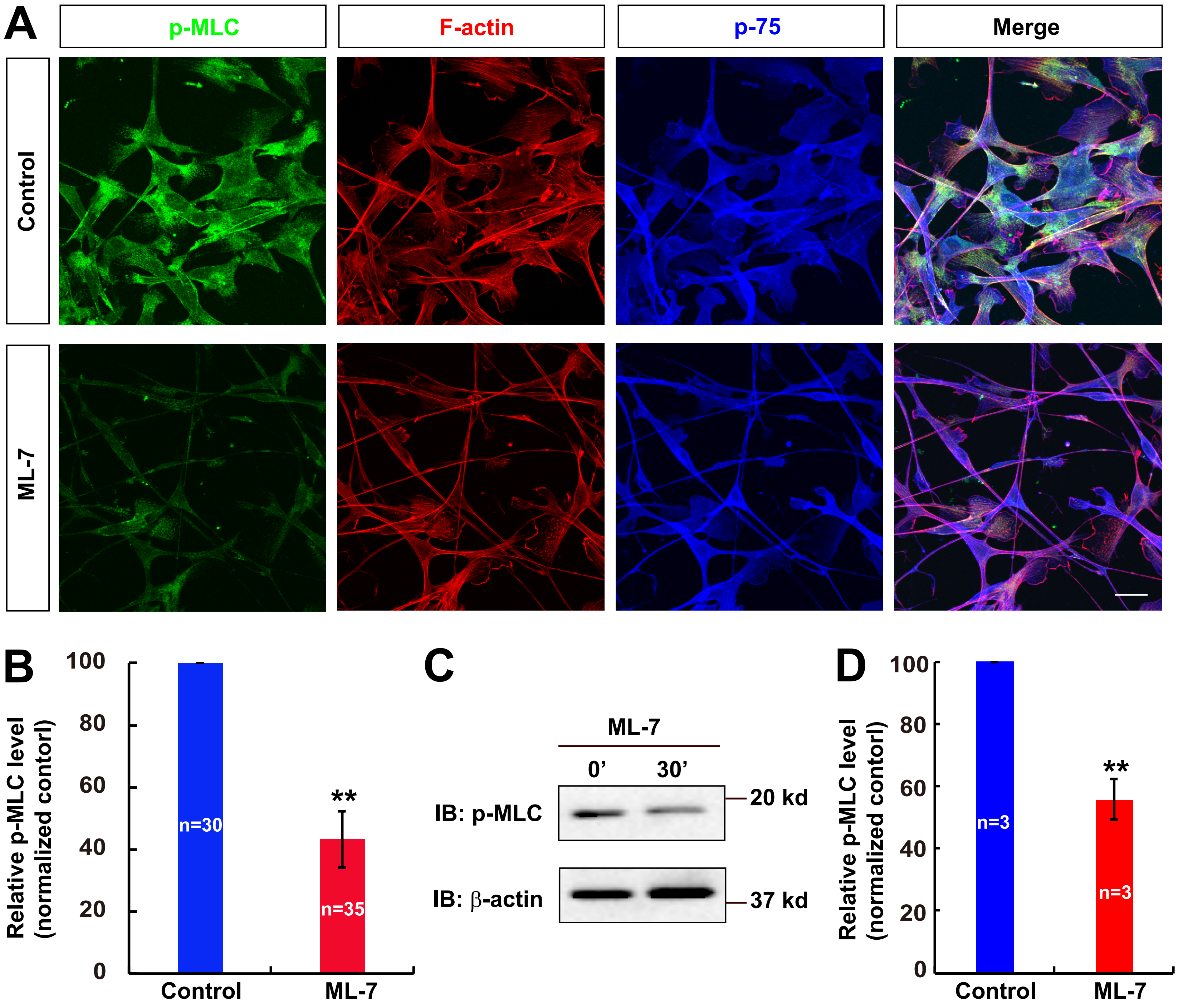
A
p-MLC
F-actin
p-75
Merge
Control
ML-7
B
Relative p-MLC level
(normalized contorl)
100
80
60
40
20
0
n=30
n=35
**
Control
ML-7
C
ML-7
0'
30'
IB: p-MLC
IB: β-actin
20 kd
37 kd
D
Relative p-MLC level
(normalized contorl)
100
80
60
40
20
0
n=3
n=3
**
Control
ML-7
